# Is the algorithm used to process heart rate variability data clinically relevant? Analysis in male adolescents

**DOI:** 10.1590/S1679-45082016AO3683

**Published:** 2016

**Authors:** Antonio Henrique Germano Soares, Breno Quintella Farah, Gabriel Grizzo Cucato, Carmelo José Albanez Bastos, Diego Giulliano Destro Christofaro, Luiz Carlos Marques Vanderlei, Aluísio Henrique Rodrigues de Andrade Lima, Raphael Mendes Ritti-Dias

**Affiliations:** 1 Universidade de Pernambuco, Recife, PE, Brazil.; 2 Hospital Israelita Albert Einstein, São Paulo, SP, Brazil.; 3 Universidade Estadual Paulista “Júlio de Mesquita Filho”, Presidente Prudente, SP, Brazil.; 4 Universidade de São Paulo, São Paulo, SP, Brazil.

**Keywords:** Autonomic nervous system, Adolescent, Heart rate/physiology, Cardiovascular system

## Abstract

**Objective:**

To analyze whether the algorithm used for the heart rate variability assessment (fast Fourier transform *versus* autoregressive methods) influenced its association with cardiovascular risk factors in male adolescents.

**Methods:**

This cross-sectional study included 1,152 male adolescents (aged 14 to 19 years). The low frequency, high frequency components (absolute numbers and normalized units), low frequency/high frequency ratio, and total power of heart rate variability parameters were obtained using the fast Fourier transform and autoregressive methods, while the adolescents were resting in a supine position.

**Results:**

All heart rate variability parameters calculated from both methods were different (p<0.05). However, a low effect size (<0.1) was found for all parameters. The intra-class correlation between methods ranged from 0.96 to 0.99, whereas the variation coefficient ranged from 7.4 to 14.8%. Furthermore, waist circumference was negatively associated with high frequency, and positively associated with low frequency and sympatovagal balance (p<0.001 for both fast Fourier transform and autoregressive methods in all associations). Systolic blood pressure was negatively associated with total power and high frequency, whereas it was positively associated with low frequency and sympatovagal balance (p<0.001 for both fast Fourier transform and autoregressive methods in all associations). Body mass index was negatively associated with high frequency, while it was positively associated with low frequency and sympatovagal balance (p values ranged from <0.001 to 0.007).

**Conclusion:**

There are significant differences in heart rate variability parameters obtained with the fast Fourier transform and autoregressive methods in male adolescent; however, these differences are not clinically significant.

## RESUMO

**Objetivo:**

Analisar se o algoritmo usado para avaliação da variabilidade da frequência cardíaca (transformada rápida de Fourier *versus* autoregressivo) influencia em sua associação com fatores de risco cardiovascular adolescentes do gênero masculino.

**Métodos:**

Estudo transversal, que incluiu 1.152 adolescentes do gênero masculino (14 a 19 anos). Componentes de baixa e alta frequência (absolutos e unidades normalizadas), razão componente de baixa frequência/componente de alta frequência e poder total da variabilidade da frequência cardíaca foram obtidos em repouso, na posição supina, usando os métodos transformada rápida de Fourier e autorregressivo.

**Resultados:**

Todos os parâmetros da variabilidade da frequência cardíaca para ambos os métodos foram diferentes (p<0,05). Entretanto, um pequeno tamanho do efeito (<0,1) foi observado para todos os parâmetros. Os coeficientes de correlação intraclasse entre os métodos variaram de 0,96 a 0,99, enquanto os coeficientes de variação foram de 7,4 a 14,8%. A circunferência abdominal foi negativamente associada com o componente de alta frequência, e positivamente associada com o componente de baixa frequência e o balanço simpatovagal (p<0,001 para a transformada rápida de Fourier e o autorregressivo em todas as associações). A pressão arterial sistólica foi negativamente associada com o poder total e o componente de alta frequência, enquanto foi positivamente associada com o componente de baixa frequência e o balanço simpatovagal (p<0,001 para a transformada rápida de Fourier e o autorregressivo em todas as associações). O índice de massa corporal foi negativamente associado com o componente de alta frequência, enquanto foi positivamente associado com o componente de baixa frequência e o balanço simpatovagal (valores de p variando de <0,001 a 0,007).

**Conclusão:**

Houve diferenças significantes nos parâmetros da variabilidade da frequência cardíaca obtidos com os métodos transformada rápida de Fourier e autorregressivo em adolescentes masculinos, mas essas diferenças não foram clinicamente significativas.

## INTRODUCTION

The autonomic nervous system, through its sympathetic and parasympathetic branches, plays a pivotal role in the control and regulation of biological functions, especially in cardiovascular system.^(1)^ In general, conditions characterized by increased sympathetic and decreased parasympathetic modulation on the heart are associated with an increased risk for cardiovascular events; whereas increased parasympathetic modulation has a cardioprotective effect.^(2)^

It is well established that spectral analysis of consecutive heartbeats, the heart rate variability (HRV), is a useful tool for assessment of the cardiac autonomic modulation^(2)^ and an important marker of cardiovascular risk.^(2-4)^ In fact, lower HRV indicates reduced parasympathetic and increased sympathetic modulations on the heart, which is associated with some conditions, including cardiovascular diseases and diabetes.^(4-6)^ Furthermore, lower HRV is associated with different cardiovascular risk factors, such as abdominal obesity, high blood pressure, and physical inactivity^(7-9)^ even in adolescence, an important phase for early identification of changes in cardiovascular control.^(10)^

The HRV spectral analysis is commonly assessed by different algorithms, such as fast Fourier transform (FFT) and autoregressive (AR) model,^(11,12)^ which provide quantification of the low frequency (LF) and high frequency (HF) oscillations of the heartbeats that can be considered representative of the sympathetic and parasympathetic modulations, respectively.^(2)^ Some studies demonstrated that HRV variables obtained by FFT and AR provided different values in HRV indicators, which were reported in different conditions (*i.e*. at rest and orthostatic stress).^(13-16)^ However, besides these aspects, it is unclear whether the differences between methods for HRV processing are clinically significant.

Thus the aim of this study was to analyze whether the algorithm used for the HRV assessment (FFT *versus* AR) influences its association with cardiovascular risk factors in a large cohort of male adolescents. We hypothesized that although having different and disagreed values, FFT and AR result in similar associations between HRV parameters and cardiovascular risk factors in this population.

## OBJECTIVE

To analyze whether the algorithm used for the heart rate variability assessment (fast Fourier transform *versus* autoregressive) influences its association with cardiovascular risk factors in a large cohort of male adolescents.

## METHODS

This is a cross-sectional study protocol and was approved by the Ethical Committee of the *Universidade do Pernambuco* in compliance with the Brazilian National Research Ethics System Guidelines and with the Declaration of Helsinki, under protocol CAAE: 0158.0.097.000.10. The target population was limited to high school students aged between 14 and 19 years. The sample comprised students from the state-owned schools in the state of Pernambuco, Brazil.

Volunteers with known *diabetes mellitus*, cardiovascular disease, and neurological or mental disabilities were excluded. Consumption of caffeinated beverages 12 hours prior to the HRV evaluation; use of alcohol, any form of tobacco, and/or other illicit drugs; and participation in any physical exercise training 24 hours before evaluation also composed the exclusion criteria. Thus, 1,152 male adolescents were included in this study.

### Demographic data, obesity indicators and blood pressure

Age, race, and place of residence were obtained using a questionnaire, and weight and height were assessed by means of automatic scales and stadiometer. The body mass index was calculated, and overweight status was determined by a body mass index above the 85th percentile for the participants’ age.^(17)^ Blood pressure was measured by oscillometer method using the Omron HEM 742 (Omron, Shangai, China), which was validated for adolescents.^(18)^ All blood pressure measurements were performed in triplicate, on the right arm placed at heart level, with the participant resting in a seated position for 5 minutes, and legs uncrossed. For the analysis, the mean value of the last two measurements was used, and high blood pressure was defined as systolic and/or diastolic blood pressure equal to or higher than the reference gender-, age-, and height-specific 95th percentile.^(19)^

### Heart rate variability analysis

HRV was assessed from the RR intervals obtained by a heart rate monitor (Polar^®^, RS800CX , USA). The adolescents remained in a supine position with spontaneous breathing for 10 minutes, after approximately 30 minutes of resting in a quiet room, at comfortable temperature. All analyses were performed with Kubios HRV software (Biosignal Analysis and Medical Imaging Group, Joensuu, Finland) by a single evaluator blinded to the other study variables. Intraclass correlation coefficient of this evaluator ranged from 0.990 to 0.993,^(20)^ following the recommendations of the task force for HRV.

The frequency-domain parameters were analyzed using the spectral analysis of HRV. The same stationary periods of the tachogram, lasting for at least 5 minutes, were broken down into bands of LF and HF using AR, with a model order of 12 by Akaike’s information criterion, and the FFT with 50% overlap and window of 256 beats. Frequencies between 0.04 and 0.4Hz were considered physiologically significant and were divided into: LF, between 0.04 and 0.15Hz; and HF, between 0.15 and 0.4Hz. The power of each component was calculated in absolute (ms^2^) and normalized units (n.u.). Normalization consisted of dividing the power of a given spectral component by the total power, minus the very LF power, and multiplying the result by 100.^(2)^ In order to interpret the results, the components LF and HF were considered, respectively, as markers of predominant sympathetic and parasympathetic modulation on the heart; and the ratio between these bands was considered as a marker of the cardiac sympathovagal balance.^(2)^ In a subsample of 27 adolescents, the reliability of HRV measures was assessed after 1 week. Intraclass correlation coefficient (ICC) ranged from 0.68 to 0.91.^(20)^

### Statistical analysis

Paired *t*-test was used for comparison of HRV parameters using FFT and AR methods. Effect sizes were calculated to estimate the magnitude of quantitative differences between both methods. The reproducibility of HRV parameters using FFT and AR methods was performed by the ICC and coefficient of variation. Pearson correlation was used to analyze the relationship between the HRV parameters (based on FFT and AR methods) and the cardiovascular risk factors.

All statistical analyses were performed using Statistical Package for Social Science (SPSS) and GraphPad Prism, and a p value <0.05 was considered statistically significant. Data are presented as mean ± standard deviation.

## RESULTS

Table 1 shows general characteristics of the sample. Mean age of the adolescents was 16.6±1.2 years and 79.2% were from the urban area. Prevalence of high blood pressure was observed in 9.7% of the sample, while overweight were present in 16.6%.

**Table 1:** General characteristics of adolescent boys

Variables	Values
Age (years)	16.6±1.2
Weight (kg)	63.7±12.6
Height (cm)	171.6±7.1
Body mass index (kg/m^2^)	21.6±3.8
Systolic blood pressure (mmHg)	121.6±12.4
Diastolic blood pressure (mmHg)	67.8±8.6
Overweight (%)	16.6
High blood pressure (%)	9.7

Values are presented in mean ± standard deviation or relative frequency.

Table 2 displays the comparison of HRV parameters between AR and FFT methods. Significant differences between FFT and AR were observed for LF (n.u.), HF (n.u.), LF (ms^2^), HF (ms^2^), total power and LF and HF ratio (p<0.05). A low effect size (*e.g*., <0.1) was observed in the comparison between methods for all HRV parameters. In addition, the ICC’s ranged from 0.960 to 0.992, whereas the coefficient of variation ranged from 7.4 to 14.8%.

**Table 2:** Comparison of heart rate variability parameters between autoregressive and fast Fourier transform methods

HRV parameters	AR	FFT	Effect size	p value
Total power (ms^2^)	3,992±3,138	3,902±3,188	0.03	<0.001
LF (ms^2^)	1,268±1,024	1,165±1,014	0.10	0.001
HF (ms^2^)	1,377±1,424	1,352±1,429	0.02	<0.001
LF (n.u.)	52.9±15.6	51.4±16.8	0.09	<0.001
HF (n.u.)	47.1±15.6	48.6±16.8	0.09	<0.001
LF/HF ratio	1.41±1.18	1.44±1.08	0.02	0.039

HRV: heart rate variability; AR: autoregressive; FFT: fast Fourier transform; LF: low frequency; HF: high frequency; n.u: normalized units.

Associations between HRV variables (by FFT and AR) and waist circumference, body mass index, and systolic blood pressure are presented in table 3. Waist circumference was negatively associated with HF, and positively associated with LF and sympatovagal balance (p<0.001 for both FFT and AR, in all associations). Systolic blood pressure was negatively associated with total power and HF, whereas it was positively associated with LF and sympatovagal balance (p<0.001 for both FFT and AR in all associations). Body mass index was negatively associated with HF, whereas it was positively associated with LF and sympatovagal balance (p-values ranged from <0.001 to 0.007).

**Table 3:** Associations between cardiovascular risk factors and heart rate variability assessed by fast Fourier transform and autoregressive methods in adolescent’s boys

Cardiovascular risk factors	Total power (ms^2^)		LF (n.u.)		HF (n.u.)		LF/HF	
			
	FFT	AR	FFT	AR	FFT	AR	FFT	AR
Systolic BP (mmHg)	-0.114 (<0.001)	-0.115 (<0.001)	-0.171 (<0.001)	0.175 (<0.001)	-0.171 (<0.001)	-0.175 (<0.001)	0.162 (<0.001)	0.155 (<0.001)
BMI (kg/m^2^)	-0.033 (0.262)	-0.028 (0.351)	-0.113 (<0.001)	0.098 (0.001)	-0.113 (<0.001)	-0.098 (0.001)	0.098 (0.001)	0.079 (0.007)
WC (c)	-0.048 (0.102)	-0.046 (0.121)	0.126 (<0.001)	0.109 (<0.001)	-0.126 (<0.001)	-0.109 (<0.001)	0.109 (<0.001)	0.091 (0.002)

Data presented as Pearson’s correlation coefficients (p value).

LF: low frequency; HF: high frequency; FFT: fast Fourier transform; AR: autoregressive; BP: blood pressure; BMI: body mass index; WC: waist circumference; n.u: normalized units.

## DISCUSSION

The main finding of this study was that the HRV variables based on FFT and AR methods are statistically different. However, the method used for HRV analysis did not influence its association with waist circumference, systolic blood pressure and body mass index, since similar correlation coefficients were observed when comparing FFT and AR methods in male adolescents.

Different algorithms, such as FFT and AR, are commonly used to assess the spectral analysis of HRV.^(2)^ FFT is a non-parametric method that computes the real spectrum. It has a high processing speed that is highly influenced by the presence of non-stationary segments of the time series, and with overlapping and windowing to filter the power spectral density. On the other hand, the AR method performs an approximation of the real spectrum, presenting good performance in time series with a reduced number of points and smoother spectral components.^(21,22)^ Parametric methods are limited to describe abrupt changes in the spectrum, particularly in signals with a limited number of parametric curves in the spectrum. The software used for analysis limits the number of turns for the iterative AR process to 16. In this situation, it is not possible to describe narrow peaks in the spectrum by the AR method, leading to differences in the LF and HF components calculation. This effect may be exacerbated especially when peaks appear in the spectrum of well-defined periodic behavior signals.^(22)^

The association between low HRV and systolic blood pressure, body mass index, and waist circumference in adolescents has been extensively demonstrated.^(7-9)^ The novelty of the present study was that the associations between HRV and these parameters are similar when comparing HRV variables obtained by both FFT and AR methods. These findings are important because FFT and AR methods have been commonly used for HRV analysis assessment at rest^(23,24)^ and during different conditions, including pharmacologic blockades,^(12)^ orthostatic stress,^(25)^ and exercise.^(26)^ This is further important because FFT and AR provided different values for the same variable (mean difference between methods: LF=24 n.u). These results are consistent with previous studies in different populations and measurement conditions.^(13-16)^ Taken together these results suggest that differences between FFT and AR methods for HRV assessment might be due to differences in data processing but with no clinical relevance, since associations between HRV and clinical characteristics were not influenced by the method used. Further investigation is needed to confirm our results.

From a clinical point of view, lower HRV, an important marker of cardiovascular risk, is associated with cardiovascular risk factors in adolescents.^(1)^ The results of this study demonstrated that although algorithm for HRV analysis (AR and FFT) might imply in different values for the same variable, these differences are not clinically significant. This is extremely relevant in the clinical setting for providing an evidence of interpretation of the HRV variables, especially showing that both methods can be used for establishing the association between HRV indicators and cardiovascular risk factors, at least, in the male adolescents.

The main strength of the present study is the large sample size. Furthermore, we tried to control various potential confounders in the study. The methodology used to collect HRV parameters was carefully selected to include only adolescents who had gone at least 24 hours without physical exercise, or use of alcohol and cigarettes, and 12 hours without ingesting caffeine. Also methodologically relevant is the fact that the participants were required to rest for at least 30 minutes prior to data collection. Moreover, since only one investigator had been blinded to all other study variables analyzed the HRV, the results are highly reproducible and reliable. Finally, the FFT and AR methods were analyzed in the same time series of the tachogram and statistical tests.

Despite these strengths, the current study has some limitations. We only included male adolescent; therefore, we cannot generalize the data to all adolescents. Although the participants’ age was tightly controlled, we could not determine the maturational stage of the participants. We only analyzed the HRV at rest and in a supine position, and it seems unclear whether similar responses would occur on a tilt-test or in a seated position. Moreover, we used only short-term HRV parameters, and agreement between methods using a long-term analysis must be studied in the future in this population. Finally, glucose levels were not measured and whether it would influence the differences between methods need further investigation.

In summary, although HRV variables based on FFT and AR algorithms demonstrated significant differences in adolescent boys, these differences are not clinically significant.

## CONCLUSION

There were significant differences in heart rate variability parameters obtained with the fast Fourier transform and autoregressive methods in male adolescents, however, these differences are not clinically significant.
